# Editorial Correspondence

**Published:** 1878-01

**Authors:** W. C. Blalock

**Affiliations:** Barnesville, Ga.


Barnesville, Ga., Dec. 31st, 1877.

*Editor Atlanta Medical and Surgical Journal:*

I desire to contribute a mite to the cause of humanity by giving the results of trials made with hydrocyanic acid in the treatment of opium habit. My experience has been limited, but so far I have yet to find a patient that could not quit morphine while under the influence of the acid. In fact, hydrocyanic acid affects them as pleasantly as morphine, without those depressing after effects. You have only to try it to be pleased with its action.

About August 3d, 1877, I began administering the acid to this class of patients. I did so with a great deal of hesitation, knowing its powerful effect. The patient was taking two grains of morphine each day. I gave him dilute hydrocyanic acid two drops, administered in glycerine, at 7 A.M., 12 M. and 8 P.M. Should the dose not sustain him, the direction was to enlarge ½ drop; should it produce giddiness or tightness about the head, to reduce dose ½ drop. This patient got well in sixty days without any unpleasant symptoms. Some time after, an old patient of mine, whom I had tried to break of the habit by the plan of gradual reduction which I had practiced, began taking the acid in the dose of two drops three times each day. She got entirely well in one month, with no desire to return to the morphine. In November I was sent for to see a young lady who had become addicted to the morphine habit from its use in neuralgia. I found her suffering more from the morphine than disease, and simply put her on the use of the acid, the dose of two drops three times each day, with direction to enlarge or decrease, as the case might require. I remained with her twelve hours to witness the effect of the drug, which was just as pleasant as I could ask. I never saw her any more in a month, when she came to town well. I have now two other inveterate cases taking the acid with good effect, apparently, and although the number of cases treated is not sufficiently large to set it down as an infallible cure, yet I trust that you will give the remedy a trial, for to my mind we have no class of patients that demand our earnest consideration more than the opium eater.

In some cases the acid is gradually reduced toward the close of treatment, while in others it is suspended at once without inconvenience. The following is the form in which I give the acid:

| | |
|---|---|
| ℞.—Diluted hydrocyanic acid, . . | gtt. xlviij. |
| Simple syrup, . . . . | f℥ij. |
| Water, . . . . . | f℥j. |
| Red aniline, . . . . | gr. xv. |

Dose.—One teaspoonful at 7 A.M., 12 M. and 8 P.M. The red aniline serves no other purpose of course, than to give it a beautiful red color. Truly,

W. C. BLALOCK, M.D.

